# Role of Endothelin-1 and Nitric Oxide in Acute Ischemic Stroke Leptomeningeal Collateral Activation

**DOI:** 10.3390/ijms26073205

**Published:** 2025-03-30

**Authors:** Marta Iacobucci, Angela Risitano, Paolo Amisano, Irene Berto, Roberto Carnevale, Vittoria Cammisotto, Francesco Biraschi, Carlo Cirelli, Maria Teresa Di Mascio, Danilo Toni, Svetlana Lorenzano, Manuela De Michele

**Affiliations:** 1 Neuroradiology Unit, Umberto I Hospital, Department of Human Neurosciences, Sapienza University, 00185 Rome, Italy; 2 Department of Human Neurosciences, Sapienza University, 00185 Rome, Italy; 3 Stroke Unit, Emergency Department, Umberto I Hospital, Sapienza University, 00185 Rome, Italy; 4 Department of Clinical Internal Anesthesiologic and Cardiovascular Sciences, Sapienza University, 00185 Rome, Italy; 5 IRCCS Neuromed, Località Camerelle, 86077 Pozzilli, Italy; 6 Department of Medical and Surgical Sciences and Biotechnologies, Sapienza University, 00185 Rome, Italy

**Keywords:** ET-1, NO, leptomeningeal collaterals, acute ischemic stroke, reperfusion

## Abstract

Good leptomeningeal collaterals (LMCs) after large vessel occlusion (LVO) extend the time window for endovascular therapy. The mechanisms regulating LMC activation are not fully understood. The aim of this study was to investigate the potential role of two vasoactive molecules endothelin-1 (ET-1)—a vasoconstrictor agent—and nitric oxide (NO)—a vasodilator agent—in the regulation of post-stroke LMCs. Ischemic stroke patients within 6 h of LVO were included. Collateral status was assessed using the Menon scoring system based on computed tomography angiography scans. Patients were accordingly divided into three groups: poor, intermediate, and good LMCs. Recanalization was evaluated using the modified thrombolysis in cerebral infarction (mTICI) score. Serum levels of ET-1 and NO were measured at three time points: T0 (<6 h), T1 (24 h), and T2 (48 h). A total of 105 patients were enrolled (mean age 76 ± 12.8 years): 44 with good (46.2%), 36 with intermediate (37.8%), and 22 with poor LMCs (23.1%). NO values decreased, whereas ET-1 values increased from T0 to T1 in all groups of patients. No significant association was found between serum ET-1 levels and collateral status. Higher ET-1 levels at T1 correlated with poor outcome regardless of the LMC status or the degree of recanalization (*p* = 0.030). A significant linear positive correlation was revealed at T0 between high levels of ET-1 and the neutrophil count (Spearman’s rho = 0.236, *p* = 0.035). Subgroup analysis showed a significant inverse correlation at T1 between NO and the collateral score (Spearman’s rho = −0.251, *p* = 0.021). Although we observed no significant association between LMC score and serum ET-1 concentrations, at 24 h higher ET-1 serum levels were predictive of poor outcome and higher NO levels were correlated with poor collateral status. These findings may indicate an inadequate microvascular reperfusion, possibly due to ET-1-mediated vasoconstriction, neutrophil activation, and NO-mediated oxidative stress, suggesting their potential role in the no-reflow phenomenon.

## 1. Introduction

In the past 10 years significant progress has been made in the treatment of acute ischemic stroke (AIS). This progress has stemmed primarily from advances in scientific knowledge of the pathophysiology of brain ischemia and a deeper comprehension of infarct progression over time. The ischemic penumbra refers to the hypoperfused, but yet viable, tissue that relies on the activation of collateral circulation and its persistence over time. Recently, alongside the still-valid concept of “time is brain”, two other clock concepts have been established: the “collateral-clock” [1] and the “clot-clock” [2] concepts. The collateral-clock refers to the persistence in certain individuals of collateral circulation, which plays a critical role in sustaining the ischemic penumbra. Thanks to collaterals the therapeutic time window has been extended to 24 h from stroke onset [3,4]. The clot-clock refers to the evolution of the clot inside the blood vessel. Over time, the occluding thrombus changes and becomes not more lysable from thrombolytic agents and less removable with endovascular procedures [2]. Patients with poor collateral circulation, as shown in multiphasic computed tomography angiography (CTA) studies, have a worse clinical outcome, even if revascularized within 6 h, compared with patients with good collateral circulation who are revascularized beyond this time window (“fast and slow progressors”) [5]. In other cases, the arterial revascularization is not followed by an efficacious microvascular reperfusion, leading to the so-called “futile recanalization” [6]. Understanding the mechanisms underlying collateral activation after ischemic stroke is crucial in developing potential therapies to sustain and improve collateral circulation, enabling endovascular treatment for more patients affected by stroke. In addition to the circle of Willis, which represents a valuable anastomotic ring at the base of the brain connecting the left and right carotid arteries and the anterior and posterior brain circulation, leptomeningeal collaterals (LMCs) represent an important network of small arteriole-to-arteriole bypass vessels that connect the anterior, middle, and posterior cerebral arteries (ACA, MCA, and PCA) in the pia mater [7].

The extent of collateral circulation varies greatly among individuals, particularly with respect to the circle of Willis. There are substantial differences in the extent and completeness of intracranial circulation in normal individuals. Collaterogenesis seems to have a genetic basis. Allelic variants of Rab GTPase-effector binding protein 2 (Rabep 2), in the Dce1 locus on chromosome 7 account for about 80% of the anatomical variation of LMCs in mice. Rabep 2 is involved in VEGF A-VEGFR 2 signaling, and its absence leads to impaired embryonal collateral formation [8]. LMCs are involved in normal brain function, dilating in response to activity in specific brain areas [9]. In pathological conditions, the recruitment of inactive collaterals can occur rapidly: for example, maximum dilation of leptomeningeal arteries occurs within 12 s after occlusion of the common carotid artery [10]. Pressure gradients between the various arterial territories involved are thought to activate LMCs [11]. However, the degree and persistence of LMC activation varies significantly between individuals [9,11]. The reasons for this individual variability and the precise mechanisms for LMC activation remain incompletely understood. Anatomic, metabolic, and hemodynamic factors have been proposed, and clinical studies have shown that poor collaterals correlate with older age, metabolic syndrome, hyperuricemia, and chronic hypertension [11].

Nitric oxide (NO) and endothelin-1 (ET-1) are two pleiotropic molecules with an opposite effect on the smooth muscle cells contraction involved in autoregulation of cerebral blood flow (CBF). NO is a potent gaseous, lipophilic vasodilator produced by NO synthase (NOS), which also has a variety of other actions [12]. NOS is present in nature in three isoforms: neuronal-NOS (nNOS), endothelial-NOS (eNOS), and inducible-NOS (iNOS). The first two isoforms are constitutively expressed in various mammalian cell types and produce NO in response to elevated intracellular calcium concentrations. In contrast, iNOS activity is independent of calcium levels and is regulated by the activation of transcription factors in response to cytokines and growth factors. NO production by iNOS is delayed and more abundant and sustained, and is thought to be primarily responsible for neurotoxic post-ischemic brain injury [13]. The massive production of reactive oxygen and nitrogen species in neurons due to NOS uncoupling contributes to neurotoxicity. In contrast, eNOS-induced NO increases immediately after stroke and seems to play a neuroprotective role through vasodilation, inhibiting platelet aggregation, and preventing leukocyte adhesion and migration, thereby limiting neuronal damage [14].

NO induces vasodilation by activating guanylate cyclase, which increases cyclic guanosine monophosphate (cGMP)-dependent protein kinase in smooth muscle cells. Soluble guanylate cyclase deficiency in mice impairs the vascular response to NO and is associated with larger infarct sizes and worse neurological outcomes during reperfusion after transient cerebral ischemia, suggesting that cGMP regulates CBF during reperfusion [15].

Additionally, NO exerts its vasoactive function by inhibiting the intracellular biosynthesis of the potent vasoconstrictor endothelin-1 (ET-1), reducing the release of soluble endothelin converting enzyme-1 (ECE-1) from endothelial cells. ECE-1 cleaves ET-1 precursor, BigET-1, and is considered the rate-limiting step in the production of ET-1 [16,17]. The potent vasoconstrictor effect of ET-1 on smooth muscle cells can be reversed by NO donors in epicardial arteries, which also inhibits ET-A receptor-binding [18,19].

The primary aim of this study was to investigate, in vivo in humans, how two key molecules, NO and ET-1, may influence the activation of LMCs following large vessel occlusion (LVO) AIS. Secondary objectives were to (1) evaluate the NO and ET-1 serum level time courses after AIS, (2) investigate the potential relationship between NO and ET-1 serum levels and the degree of recanalization, and (3) examine the correlation between NO and ET-1 serum levels and clinical outcomes.

## 2. Results

We enrolled 105 patients with LVO AIS, 39 males (37.1%) and 66 females (62.9%); mean ± SD age 76.0 ± 12.8 years. Thirty patients (28.5%) had a wake-up stroke or unknown onset of symptoms.

### 2.1. Patients’ Characteristics and Clinical Outcome

We did not find any statistically significant differences between the three groups as regards demographic characteristics, previous medical therapy, blood pressure, heart rate, or serum glucose values at the three time points (Table 1, Supplementary Tables S1 and S2). Patients with poor collateral status had a more severe stroke with higher NIHSS score at T0 (poor vs. intermediate vs. good collateral status group, *p* = 0.035). Clinical outcome expressed by mRS at 90 days post-stroke was not influenced by the collateral status (Supplementary Table S3).

### 2.2. Biomarkers

**Figure 1:**
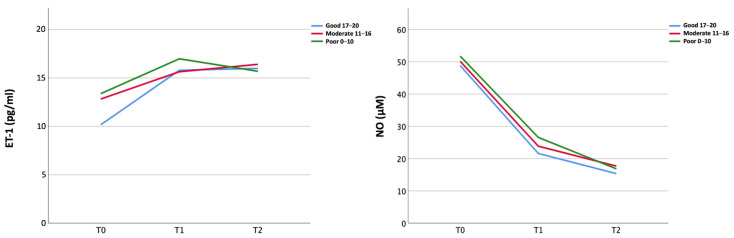
Temporal course of peripheral biomarkers (ET-1 and NO) in the three collateral groups.

ET-1 serum level increased whereas NO serum levels decreased from T0 to T1 in the overall population (ET-1 11.93 pg/mL vs. 16.63 pg/mL; NO 49.71 μM vs. 23.46 μM) regardless of the collateral status (Figure 1). ET-1 levels reached a plateau from T1 and T2, while NO levels showed a slight further decrease from T1 to T2 (Figure 1, Supplementary Tables S4 and S5).

**Figure 2:**
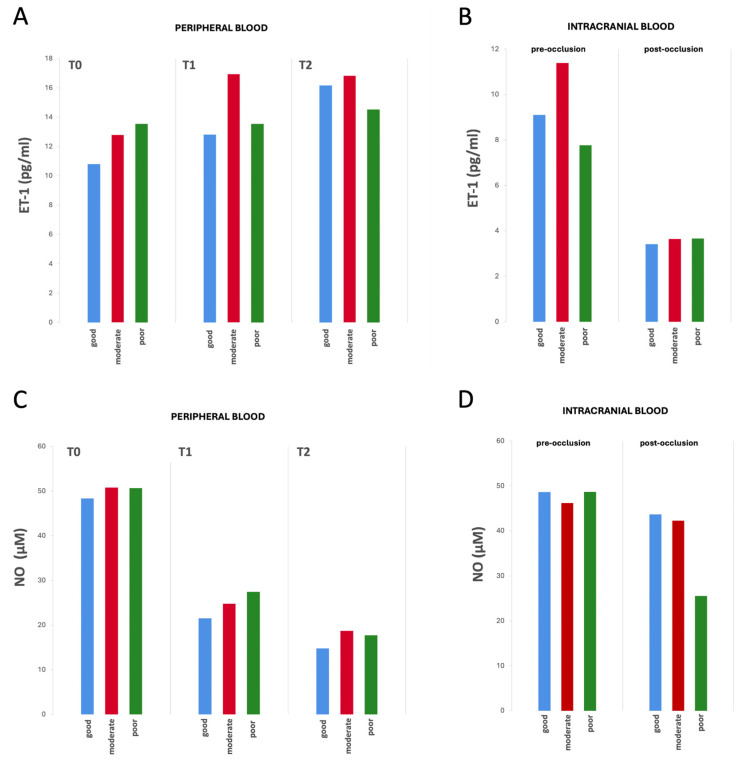
Differences in biomarker values from peripheral and intracranial blood by collateral status over time (from T0 to T2). (**A**) ET-1 values from peripheral blood samples, (**B**) ET-1 values from intracranial blood samples (pre- and post-occlusion), (**C**) NO values from peripheral blood samples, and (**D**) NO from intracranial blood samples (pre- and post-occlusion). No statistically significant differences were observed in ET-1 or NO concentrations between the three groups at any of the analyzed time points. Mean biomarker concentrations for each group and time point are reported in Supplementary Tables S4 and S5.

The biomarker values—both in the peripheral venous and pre- and post-occlusion arterial intracranial blood samples—examined at the three time points did not show significant differences between groups according to the collateral status (Figure 2, Supplementary Tables S4 and S5).

Patients with higher ET-1 levels at T1 had poor outcome (mRS at 3 months) regardless of the LMC status and the degree of recanalization (Table 2).

**Figure 3:**
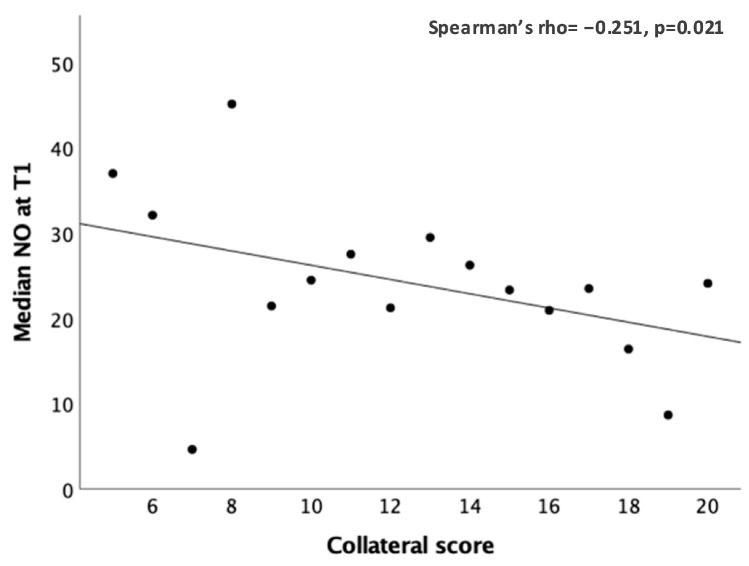
Significant correlation between the peripheral NO values (μM) and the collateral score at T1.

Subgroup analysis showed a significant inverse correlation at T1 between NO and the collateral score (Spearman’s rho = −0.251, *p* = 0.021; Figure 3).

**Figure 4:**
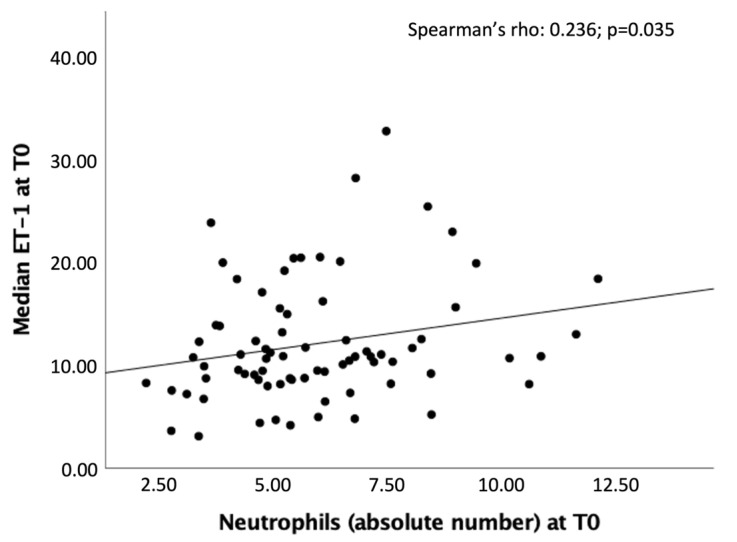
Significant correlation between the peripheral ET-1 values (pg/mL) and the neutrophil count at T0.

A significant linear positive correlation was found at T0 between high levels of ET-1 and neutrophil count (Spearman’s rho = 0.236, *p* = 0.035) (Figure 4).

We did not find any statistically significant differences in the biomarkers levels between patients receiving either IVT, MT, or both treatments.

## 3. Discussion

The autoregulation of cerebral arteries and the neurovascular coupling remains a highly complex and not fully elucidated phenomenon [20]. The primary goal of this is to maintain stable cerebral blood flow (CBF) across the brain, adjusting dynamically to variations in metabolic demand. The small cerebral arteries, arterioles, venules, and capillaries are essential for preserving blood flow within the brain. During ischemic conditions, the initial defense mechanism is the vasodilation of cerebral arterioles and the activation of LMCs, which help to support the ischemic penumbra [11] until recanalization. A thorough understanding of the mechanisms underlying LMC activation is imperative. In fact, this knowledge is pivotal for the development of novel therapeutic strategies aimed at preserving the ischemic penumbra, thereby optimizing recovery following recanalization interventions.

The main aim of this study was to investigate the role of ET-1 and NO in post-LVO stroke LMC activation in vivo in humans. We did not find a statistically significant correlation between serum concentrations of ET-1 and the extent of LMCs, suggesting that their activation is a complex, multifactorial process influenced by multiple interacting factors.

We found an opposite time course of ET-1 and NO after AIS. The ET-1 increased while NO decreased from T0 to T1, regardless of LMC status. A plausible explanation is that NO plays a key role in LMC activation, with higher concentrations in the early phases of stroke to promote collateral vasodilatation, followed by a progressive decline; on the contrary, ET-1 levels rose over the first 24 h, likely as a counterregulatory response. Indeed, there is evidence that ET-1 and NO directly regulate each other to achieve vascular-tone homeostasis. Stimulation of NO production in endothelial cells can reduce ET-1 expression and production [12]. We can hypothesize that, following the rapid response of the vascular endothelium to reduced CBF —characterized by decreased ET-1 and increased NO [21] in order to activate LMCs— there may be a subsequent decrease in peripheral NO levels and a concurrent rise in ET-1, as observed in our study. In fact, ET-1 exerts its vasoconstrictive action mainly through the ETA receptors widely represented on the smooth muscle cells of brain arterioles and its increase, if not counteracted by the vasodilator effect of NO, could lead to vasoconstriction of brain arterioles and poor reperfusion [22].

Moreover, we observed that higher NO levels at 24 h were associated with baseline poorer collateral status. This may be explained by the hypothesis that increased NO production in patients with limited leptomeningeal collaterals serves as a compensatory mechanism, potentially enhancing the activation and vasodilation of underdeveloped collateral pathways to better support the ischemic penumbra. Another possible explanation could be that at 24 h, the vasodilator effect of NO is overcome by the deleterious nitrosative production of free radicals and neurotoxic effects. In endothelial cell dysfunction, eNOS expression increases and the enzyme becomes uncoupled, producing the highly reactive superoxide species instead of NO [13]. We also found that higher ET-1 levels at 24 h correlated with poor outcome regardless of the LMC status and the degree of recanalization, suggesting that this may be attributable to its impact on the microcirculation.

Indeed, we observed a positive correlation between ET-1 levels and neutrophil counts at baseline, which also supports the role of ET-1 in post-stroke neuroinflammation. Several studies have shown that ET-1 enhances neutrophil adhesion to endothelial cells, promotes the production of chemotactic factors, and activates specific endothelin receptors (ETB), all of which contribute to the inflammatory response and neutrophil recruitment, particularly in ischemic injury [23,24,25,26]. This sequence of events could have toxic effects on the microvasculature after LVO recanalization, leading to incomplete reperfusion of the ischemic brain tissue (the no-reflow phenomenon), ultimately resulting in a poor outcome [6].

This is further supported by previous studies in humans that have observed a positive correlation between elevated ET-1 levels and unfavorable outcomes [27,28]. However, other studies have reported normal ET-1 levels following AIS [29,30,31,32]. These conflicting results may be due to the different timing of sampling across studies: in the early stage of stroke (within 24 h after the onset of symptoms), ET-1 levels were increased, as in our study, whereas they were reported to be normal if the sampling was performed after 24 h. To the best of our knowledge, this is the first study investigating the relationship between ET-1 and collateral activation following AIS.

The primary limitation of our study was the small sample sizes of subgroups, which may have limited our ability to detect statistically significant differences in the association between collateral status and some of the examined biomarkers.

In addition, some considerations need to be made. First, LMC activation is very fast after LVO; it has been demonstrated in animals that the leptomeningeal arteries reach their maximum dilation within 12 s following the occlusion of the common carotid artery [10]. T0 in our study represented the admission time at the emergency room which was variable within 80 min and 24 h from stroke onset. It is possible that the concentrations we detected in the peripheral and intracranial blood were only a fair surrogate of what could be found in the brain during the first minutes after LVO. Second, NO is a gaseous lipophilic molecule not measurable in the blood. We determined the metabolites of NO (nitrites and nitrates), and this could have partly invalidated the study results. Third, NO is produced by different NOS isoforms: the endothelial, which acts as a powerful vasodilator, and the neuronal and the inducible NOS, which all have different actions [12,13]. Unfortunately, we could not distinguish the metabolites of NO to determine which isoforms they are generated from. Moreover, the biochemistry of NO is highly complex, with new insights into the regulation of NO biosynthesis and the mechanisms of signal transduction continuously emerging [12].

Finally, the single-center design of the project may have limited the generalizability of our findings.

The strengths of this study lay in a comprehensive biomarker analysis of both peripheral and intracranial blood samples and its integration with neuroimaging techniques, a specific focus on clinically relevant condition such as leptomeningeal collateral activation after AIS, and the prospective design.

In conclusion, although our results did not demonstrate a direct correlation between ET-1/NO levels and the degree of collateral status at baseline, it was not possible to exclude the potential role of these vasomotor agents in LMC activation. Further investigations in order to study the vasoactive effects of these molecules on collaterals activation in experimental middle cerebral artery occlusion are needed. In our population, higher ET-1 at admission correlated with neutrophil count, and at 24 h it predicted a poor 3-month outcome, whereas an inverse correlation between NO and the collateral score was observed at 24 h. These findings may suggest a novel hypothesis about the involvement of these molecules in the no-reflow phenomenon. Further explorations could provide valuable insights and a better understanding of futile recanalization mechanisms.

## 4. Materials and Methods

### 4.1. Patients

This prospective, observational study included consecutive adult patients (aged ≥18 years) diagnosed with AIS. Enrollment was carried out at the Emergency Department of our university hospital from November 2019 to December 2021.

Eligible participants met the following inclusion criteria: presentation within six hours of symptom onset or with wake-up stroke and evidence of LVO affecting the anterior circulation, including tandem occlusions involving the internal carotid artery [ICA] plus the middle cerebral artery [MCA], M1, or proximal M2 segments, as confirmed by multiphasic computed tomography angiography (CTA).

Exclusion criteria were: presence of hemorrhagic stroke, AIS without LVO, and contraindications to iodinated contrast agents.

At admission, all patients underwent comprehensive general and neurological evaluations, including electrocardiography (ECG). Data collected comprised demographic characteristics and vascular risk factors, such as hypertension, atrial fibrillation, diabetes mellitus, hypercholesterolemia, significant carotid stenosis, tobacco use, alcohol consumption, prior transient ischemic attack (TIA), previous stroke or myocardial infarction, renal impairment, oncological disease, and current pharmacological therapies.

Functional status was assessed using the modified Rankin Scale (mRS), both pre-stroke and at three months following stroke onset, to determine clinical outcomes. Stroke subtype classification was performed according to the Trial of Org 10,172 in Acute Stroke Treatment (TOAST) criteria [33].

Three distinct time points were defined: time 0 at admission (T0), time 1 (T1) at 24 h, and time 2 (T2) at 48 h. At each of these intervals, stroke severity was evaluated using the National Institutes of Health Stroke Scale (NIHSS), and blood samples were collected. Additionally, physiological parameters, including blood glucose, arterial pressure, heart rate, and body temperature, were measured at the same time points.

### 4.2. Laboratory Data

Peripheral venous blood samples were collected into tubes containing 3.8% sodium citrate anticoagulant or without additives. Samples were processed by centrifugation at 300× *g* for 10 min at room temperature to separate plasma and serum. Aliquots were then stored at −80 °C for 6 months from collection, pending analysis.

Routine laboratory parameters were assessed at T0, including complete blood count, C-reactive protein (CRP), creatinine, glucose, and lipid profile. Selected parameters (blood count, CRP, and glucose) were re-evaluated at T1 and T2.

Intracranial blood samples were obtained during endovascular thrombectomy, using a microcatheter positioned (1) proximal to the occlusion site, prior to reperfusion (pre-occlusion sample), and (2) distal to the occlusion, following recanalization (post-occlusion sample).

#### 4.2.1. ET-1

Serum concentrations of ET-1 were quantified by enzyme-linked immunosorbent assay (ELISA) utilizing a commercially available kit (TEMA Ricerca srl, Castenaso, Bologna, Italy), following the manufacturer’s instructions. Results are expressed in pg/mL. The intra- and inter-assay coefficients of variation were consistently below 10%.

#### 4.2.2. NO

Due to the intrinsic instability and brief half-life of nitric oxide (NO)—approximately six seconds—its measurement was indirectly performed by quantifying stable metabolic by-products, nitrite (NO_2_^−^) and nitrate (NO_3_^−^), collectively referred to as NOx [34].

NOx levels were assessed in serum samples using a colorimetric assay kit (Abcam, Cambridge, UK). A volume of 100 µL of sample was incubated under constant stirring at 37 °C for 10 min. Concentrations were expressed in µM. The intra- and inter-assay coefficients of variation were 2.9% and 1.7%, respectively.

### 4.3. Radiological Data

At admission, all patients underwent multiphasic CTA to confirm LVO and to assess collateral circulation, which determined eligibility for endovascular intervention. In cases of wake-up or unknown time of onset stroke, additional CT or MR perfusion imaging was performed; however, inclusion in the study was contingent upon multiphasic CTA availability.

Collateral circulation was evaluated using the regional leptomeningeal collateral (rLMC) scoring system, according to the method described by Menon et al. [35]. This semiquantitative scale assesses retrograde collateral flow distal to the occluded MCA or intracranial ICA, relative to the contralateral hemisphere. Scoring was assigned to six MCA regions, the ACA territory, and the basal ganglia. Each region was graded as follows: 0 points for absence of collateral vessels, 1 point for diminished vessel prominence, and 2 points for equal or increased prominence compared to the healthy side.

Particular emphasis was placed on collateral vessels in the Sylvian fissure, with scores of 0, 2, or 4 points reflecting absent, reduced, or equal/increased prominence, respectively. The total collateral score ranged from 0 to 20. Based on total scores, collateral circulation was classified as good (17–20), intermediate (11–16), or poor (≤10).

Non-contrast CT scans were analyzed to calculate the Alberta Stroke Program Early CT Score (ASPECTS) [36].

Eligible patients underwent digital subtraction angiography (DSA), and reperfusion success was graded using the modified Treatment in Cerebral Infarction (mTICI) scale: 0 (no perfusion), 1 (minimal perfusion), 2a (reperfusion < 50% of the affected territory), 2b (reperfusion ≥ 50%), and 3 (complete reperfusion) [37].

For analytical purposes, recanalization outcomes were dichotomized as poor (mTICI 0–2a) or successful (mTICI 2b–3).

Final infarct volumes were measured using diffusion-weighted MRI (DWI) performed 24–48 h post-procedure, employing the ABC/2 method [38]. In cases of MRI contraindications, non-contrast CT was used for follow-up imaging.

Three experienced neuroradiologists independently reviewed all imaging studies, including LMC scores, ASPECTS, and infarct volumes. Any discrepancies were resolved through consensus discussion, with the involvement of a fourth neuroradiologist in cases where initial agreement was not achieved.

### 4.4. Statistical Analysis

Descriptive statistics were applied to summarize the characteristics of the overall cohort and the three collateral circulation subgroups. Continuous variables were reported as means or medians, depending on distribution normality, while categorical variables were presented as absolute and relative frequencies.

Group comparisons for categorical variables were performed using Fisher’s exact test or the chi-square test, as appropriate. Continuous variables with normal and non-normal distributions were compared using the *t*-test for independent samples or the Mann–Whitney U test, respectively. When comparing more than two groups, a one-way ANOVA was performed, and for statistically significant results, appropriate post hoc analyses were conducted using Tukey’s test when equal variances were assumed, or the Games–Howell test when this assumption was violated.

Correlations between variables were assessed via Pearson’s or Spearman’s correlation coefficients, based on data distribution. Temporal trends of molecular biomarkers were analyzed across predefined time points.

A two-tailed *p*-value < 0.05 was considered indicative of statistical significance. All analyses were conducted using SPSS software (IBM Corp., SPSS Statistics for Windows, Version 25.0, Armonk, NY, USA).

## Figures and Tables

**Table 1:** Demographics and clinical characteristics in the overall study population and by collateral status.

	All Patients n = 105	Good n = 44	Moderate n = 36	Poor n = 22	*p*-Value
**Demographics and Clinical Characteristics**					
Age (years), mean (SD)	76.0 (12.8)	75.0 (11.1)	77.1 (14.9)	77.8 (10.1)	0.604
Sex (females) (%)	66 (62.9)	32 (72.7)	20 (55.6)	13 (59.1)	0.248
Pre-stroke mRS (%)					
- 0	77 (73.3)	38 (86.4)	23 (63.9)	14 (63.6)	0.132
- 1	8 (7.6)	2 (4.5)	3 (8.3)	3 (13.6)	
- 2	6 (5.7)	3 (6.8)	3 (8.3)	0	
- 3	11 (10.5)	1 (2.3)	6 (16.7)	3 (13.6)	
- 4	2 (1.9)	0	1 (2.8)	1 (4.5)	
- 5	1 (1.0)	0	0	1 (4.5)	
Pre-stroke mRS 0–1 (%)	85 (81)	40 (90.9)	26 (72.2)	17 (77.3)	0.087
Obesity (%)	16/100 (16.0)	8/42 (19.0)	3/34 (8.8)	5/21 (23.8)	0.291
Smoking (%)	16/99 (16.2)	8/43 (18.6)	3/33 (9.1)	5/20 (25.0)	0.289
Alcohol consumption (%)	6/104 (5.8)	1 (2.3)	4/35 (11.4)	1 (4.5)	0.221
Drug abuse (%)	3/104 (2.9)	0	2/35 (5.7)	1 (4.5)	0.294
Hypertension (%)	84/103 (81.6)	33/43 (76.7)	30/35 (85.7)	19 (86.4)	0.493
Hyperlipidemia (%)	56/100 (56.0)	29/43 (67.4)	17/34 (50.0)	8/20 (40.0)	0.089
Atrial fibrillation (%)	63/102 (61.8)	25/43 (58.1)	20/35 (57.1)	18/21 (85.7)	0.060
Ischemic cardiopathy (%)	32/104 (30.8)	10 (22.7)	11/35 (31.4)	9 (40.9)	0.301
Diabetes mellitus (%)	25/104 (24.0)	8 (18.2)	11/35 (31.4)	6 (27.3)	0.381
Previous stroke (%)	18/102 (97.1)	5 (11.4)	8/34 (23.5)	4/21 (19.0)	0.357
Previous TIA (%)	8/103 (7.8)	2 (4.5)	5/35 (14.3)	1/21 (4.8)	0.236
Previous CEA (%)	4/104 (3.8)	1 (2.3)	3/35 (8.6)	0	0.203
Carotid artery stenosis < 50%	35/103 (34.0)	15/43 (34.9)	13/35 (37.1)	7 (31.8)	0.919
Carotid artery stenosis (50–70%)	7/103 (6.8)	4/43 (9.3)	3/35 (8.6)	0	0.343
Carotid artery stenosis > 70%	15/104 (14.4)	4/43 (9.3)	5/35 (14.3)	6 (27.3)	0.146
**Stroke Characteristics**					
Stroke onset on	30/104 (28.8)	14 (31.8)	12/35 (34.3)	3 (13.6)	0.204
- Awakening/unknown (%)					
- Stroke on awakening	22/105 (21.0)	10/44 (22.7)	9 (25.0)	2 (9.1)	0.312
NIHSS, median (IQR)					
- Admission (T0)	15 (10–20)	14 (10–20)	15 (9–17)	18 (14–22.50)	**0.035**
- 24 h (T1)	10 (4–17)	8 (2–14)	10.50 (5.50–20)	14 (10–17.25)	0.090
- 48 h (T2)	9.50 (3–17)	6 (2–16)	10.50 (4–18)	13.9 (7.8)	0.095
- Discharge	5 (2–9)	4 (1–9)	5 (2–11)	5 (4.50–14.25)	0.281
ASPECTS					
- Mean (SD)	8.22 (1.32)	8.48 (1.19)	8.24 (1.23)	7.86 (1.21)	0.155
- Median (IQR)	8 (7–9)	9 (8–9)	8 (7.75–9)	8 (7–8.25)	
Vessel occlusion site (%)					
- M1	57/97 (58.8)	24/41 (58.5)	17/34 (50.0)	13/19 (68.4)	
- M2	29/97 (29.9)	12/41 (29.3)	14/34 (41.2)	3/19 (15.8)	
- ICA	2/97 (2.1)	0	1/34 (2.9)	1/19 (5.3)	
- Tandem ICA + M1	9/97 (9.3)	5/41 (12.2)	2/34 (5.9)	2/19 (19.5)	0.424
Collateral status					**<0.001**
- Mean (SD)	14.65 (4.18)	18.45 (1.0)	13.72 (1.73)	8.50 (2.33)	
- Median (IQR)	15.50 (11–18)	18 (18–19)	14 (12–15)	8.50 (6.75–10)	
Recanalization treatment (%)					
- IVT	53 (50.5)	20 (45.5)	20 (55.6)	12 (54.5)	0.622
- MT	90/104 (85.3)	38 (86.4)	32/35 (91.4)	18 (81.8)	0.562
- IVT + MT	42 (41.2)	15 (34.1)	18 (50.0)	9 (40.9)	0.355
MT technique					
- Thromboaspiration	41/80 (51.2)	17/36 (47.2)	14/28 (50.0)	10/15 (66.7)	0.202
- Stent retrieving	11/80 (13.8)	3/36 (8.3)	7/28 (25.0)	1/15 (6.7)	
- Thromboaspiration +	23/80 (28.7)	12/36 (33.3)	7/28 (25.0)	3/15 (20.0)	
- Stent retriever					
- Other	5/80 (6.3)	4736 (11.1)	0	1/15 (6.7)	
Onset to IVT time (min),	150.0	150.0	151.0	135.0	
Median (IQR)	(120–190)	(131.25–205.0)	(110–190)	(100.0–177.0)	0.635
Onset to MT time (min),	265.0	265.0	286.0	208.50	0.300
Median (IQR)	(197.50–350)	(213–347.50)	(194.50–438.50)	(177.0–332.0)	
TICI (%)					
- 0	7/83 (8.4)	1/36 (2.8)	3/30 (10.0)	3/16 (18.8)	0.090
- 1	5/83 (6.0)	0	4/30 (13.3)	0	
- 2a	11/83 (13.3)	5/36 (13.9)	5/30 (16.7)	1/16 (6.3)	
- 2b	17/83 (20.5)	10/36 (27.8)	5/30 (16.7)	2/16 (12.5)	
- 3	43/83 (51.8)	20/36 (55.6)	13/30 (43.3)	10/16 (62.5)	
TICI 2b–3 (%)	60/83 (72.3)	30/36 (83.3)	18/30 (60.0)	12/16 (75.0)	0.102
Imaging for V measurement:					
- RM (%)	85/103 (82.5)	36 (81.8)	31/35 (88.6)	17/21 (81.0)	
- CT (%)	18/103 (17.5)	8 (18.2)	4/35 (11.4)	4/21 (19.0)	0.655
Infarct volume, median	9.70	5.90	13.35	14.60	0.482
(IQR)	(3.60–20.50)	(2.80–12.25)	(5.08–20.50)	(8.85–41.80)	
Stroke etiopathogenesis (%)					
- LV atherothrombosis	10/99 (10.1)	5/41 (12.2)	1/34 (2.9)	4/21 (19.0)	0.134
- Cardioembolic	68/99 (68.7)	25/41 (61.0)	25/34 (73.5)	17/21 (81.0)	
- Other determined cause	7/99 (14.1)	4/41 (9.8)	3/34 (8.8)	0	
- Other undetermined cause	14/99 (14.1)	7/41 (17.1)	5/34 (14.7)	0	

SD = standard deviation; mRS = modified Rankin Scale; TIA = transient ischemic attack; CEA = carotid endarterectomy; NIHSS = National Institutes of Health Stroke Scale; IQR = interquartile range; T0 = time point 0; T1 = time point 1; T2 = time point 2; ASPECTS = Alberta Stroke Program Early CT Score; M1 = M1 segment of the middle cerebral artery; M2 = M2 segment of the middle cerebral artery; ICA = internal carotid artery; IVT = intravenous thrombolysis; MT = mechanical thrombectomy; TICI = thrombolysis in cerebral infarction; V = volume: RM = magnetic resonance imaging; CT = computed tomography; LV = large vessel; *p*-values highlighted in bold indicate statistically significant results (*p* < 0.05). Section headings are formatted in italics and highlighted in gray to distinguish different categories of data within the table.

**Table 2:** ET-1 values at T1 in the overall study population by clinical outcome.

	mRS 0–2 (n = 39)	mRS 3–6 (n = 61)	*p*-Value
ET-1 at T1 (pg/mL), median (IQR)	12.44 (10.61–16.58)	16.95 (11.81–23.44)	**0.017**

mRS = modified Rankin Scale; ET-1 = endothelin-1; T1 = time point 1; pg/dL = picograms per deciliter; IQR = interquartile range;. *p*-values highlighted in bold indicate statistically significant results (*p* < 0.05).

## Data Availability

Data are contained within the article and the Supplementary Materials.

## Supplementary Materials

The following supporting information can be downloaded at: https://www.mdpi.com/article/10.3390/ijms26073205/s1.
