# Supplementary material for: Role of Endothelin-1 and Nitric Oxide in Acute Ischemic Stroke Leptomeningeal Collateral Activation
Source: Int J Mol Sci. 2025 Mar 30;26(7):3205. doi: 10.3390/ijms26073205 (PMC11989326; doi:10.3390/ijms26073205)
Supplement: Supplementary file 1 [file ijms-26-03205-s001.zip › ijms-3534823-supplementary.pdf]

Supplementary Material

Supplementary Table S1. Domiciliary therapy prior to stroke in the overall study population and by collateral status

	**All Patients n = 105**	**Good n = 44**	**Moderate n = 36**	**Poor n = 22**	**p-value**
Anticoagulant (%)	26/97 (26.8)	11/41 (26.8)	8/33 (24.2)	7/21 (33.3)	
- Warfarin	8/26 (30.8)	4/11 (36.4)	2/8 (25.0)	2/7 (28.6)	
- Acenocumarol	3/26 (11.5)	0	2/8 (25.0)	1/7 (14.3)	
- Enoxaparin	5/26 (19.2)	2/11 (18.2)	2/8 (25.0)	1/7 (14.3)	0.762
- Warfarin/enoxaparin	1/26 (3.8)	0	1/11 (12.5)	0	
- Dabigatran	1/26 (3.8)	1/11 (9.1)	0	0	
- Apixaban	2/26 (7.7)	0	1/8 (12.5)	1/7 (14.3)	
- Edoxaban	2/26 (7.7)	2/11 (18.2)	0	0	
- Rivaroxaban	4/26 (15.4)	2/11 (18.2)	0	2/7 (28.6)	
Antiplatelet (%)	39/96 (40.6)	15/41 (36.6)	16/32 (50.0)	6/20 (30.0)	
- ASA	23/38 (60.5)	7/14 (50.0)	10/16 (62.5)	4/6 (66.7)	
- Clopidogrel	7/38 (18.4)	3/14 (21.4)	2/16 (12.5)	2/6 (33.3)	0.306
- ASA+clopidogrel	5/38 (13.2)	3/14 (21.4)	2/16 (12.5)	0	
- Cardirene	2/38 (5.3)	1/14 (7.1)	1/16 (6.3)	0	
- Ticlopidina	1/38 (2.6)	0	1/16 (6.3)	0	
Lipid-lowering drugs (%)	31/95 (32.6)	13/40 (32.5)	11/32 (34.4)	5/20 (25.0)	0.766
ACE-1/AREB (%)	49/96 (51.0)	16/41 (39.0)	20/32 (62.5)	12/20 (60.0)	0.096
Diuretics (%)	30/97 (30.9)	11/41 (26.8)	11732 (34.4)	8/21 (38.1)	0.623
Beta-blockers (%)	42/97 (43.3)	18/41 (43.9)	14/32 (43.8)	9/21 (42.9)	0.997
Calcium antagonists (%)	10/96 (10.4)	4/41 (9.8)	4/32 (12.5)	2/20 (10.0)	0.925
PPI (%)	31/97 (32.0)	13/41 (31.7)	12/32 (37.5)	5/21 (23.8)	0.578
Oral glucose-lowering drugs (%)	16/97 (16.5)	7/41 (17.1)	6/32 (18.8)	3/21 (14.3)	0.914
Insulin (%)	6/94 (6.4)	1/38 (2.6)	5/32 (15.6)	0	**0.035**
Nitrates (%)	6/76 (7.9)	1/30 (3.3)	3/29 (10.3)	2/16 (12.5)	0.462

ASA = acetylsalicylic acid; ACE-1/AREB = angiotensin-converting enzyme-1 inhibitor/angiotensin receptor blockers; PPI = proton-pump inhibitors.
p-values highlighted in bold indicate statistically significant results ($p < 0.05$).

Supplementary Table S2. Vital parameters in the overall study population and by collateral status at the three time points (T0, T1, T2)

	**All Patients n = 105**	**Good n = 44**	**Moderate n = 36**	**Poor n = 22**	**p-value**
SBP (mmHg), mean (SD)					
- T0	144 (28.5)	144.7 (29.1)	145.1 (25.3)	143.1 (24.6)	0.962
- T1	141.1 (22.7)	139.7 (22.8)	139.2 (21.4)	148.8 (25.3)	0.321
- T2	142.5 (21.5)	139.7 (20.5)	145.7 (20.9)	143.4 (25.5)	0.518
DBP (mmHg), mean (SD)					
- T0	79.8 (14.1)	80.6 (14.6)	78.6 (12.9)	80.4 (16.4)	0.824
- T1	76.4 (16.5)	75.7 (12.2)	75.7 (16.7)	80.1 (24.4)	0.617
- T2	78.2 (14.2)	78.2 (13.0)	78.2 (13.1)	78.6 (19.10)	0.995
HR (bpm), mean (SD)					
- T0	78.3 (15.4)	77.1 (14.7)	81.7 (15.6)	74.7 (17.0)	0.250
- T1	80.6 (18.3)	75.4 (13.8)	85.7 (17.7)	82.8 (26.5)	**0.046**
- T2	81.7 (22.2)	81.3 (21.1)	80.9 (18.8)	84.4 (30.4)	0.866
BT (°C), mean (SD)					
- T0	36.2 (0.4)	36.2 (0.4)	36.3 (0.4)	36.2 (0.3)	0.805
- T1	37.0 (0.6)	37.0 (0.6)	37.1 (0.7)	37.0 (0.6)	0.763
- T2	37.0 (0.6)	36.9 (0.5)	36.9 (0.7)	37.1 (0.6)	0.503
HGT (mg/dL), mean (SD)					
- T0	142.5 (55.3)	138.7 (49.4)	149.2 (70.2)	141.90 (37.83)	0.722
- T1	128.2 (45.6)	114.3 (30.4)	145.7 (55.7)	132.9 (47.7)	**0.025**
- T2	115.6 (30.0	110.7 (24.5)	118.8 (36.2)	121.6 (30.2)	0.423

mmHg = millimeters of mercury; SBP = systolic blood pressure; SD = standard deviation; DPB = diastolic blood pressure; bpm = beats per minute; BT = body temperature; °C = degrees Celsius; HGT = haemoglucotest; mg/dL = milligrams per deciliter;

p-values highlighted in bold indicate statistically significant results ($p < 0.05$).

Supplementary Table S3. Clinical outcome measures in the overall study population and by collateral status

	**All Patients n = 105**	**Good n = 44**	**Moderate n = 36**	**Poor n = 22**	**p-value**
mRS at 90 days (%)					
- 0	19/100 (19.0)	11/41 (26.8)	6/35 (17.1)	1/21 (4.8)	
- 1	11/100 (11.0)	3/41 (7.3)	3/35 (8.6)	5/21 (23.8)	
- 2	9/100 (9.0)	3/41 (7.3)	5/35 (14.3)	1/21 (4.8)	0.464
- 3	10/100 (10.0)	5/41 (12.2)	4/35 (11.4)	1/21 (4.8)	
- 4	17/100 (17.0)	7/41 (17.1)	5/35 (14.3)	5/21 (23.8)	
- 5	10/100 (10.0)	5/41 (12.2)	3/35 (8.6)	2/21 (9.5)	
- 6	24/100 (24.0)	7/41 (17.1)	9/35 (25.7)	6/21 (28.6)	
mRS 0–1 at 90 days (%)	30/100 (30.0)	14/41 (34.1)	9/35 (25.7)	6/21 (28.6)	0.718
mRS 0–2 at 90 days (%)	39/100 (29.0)	17/41 (41.5)	14/35 (40.0)	7/21 (33.3)	0.818
mRS 2–6 at 90 days (%)	70/100 (70.0)	27/41 (65.9)	26/35 (74.3)	15/21 (71.4)	0.718
mRS 3–6 at 90 days (%)	61/100 (61.0)	24/41 (58.5)	21/35 (60.0)	14/21 (66.7)	0.818
In-hospital death (%)	18/100 (18.0)	4/41 (9.8)	6/35 (17.1)	6/21 (28.6)	0.166

mRS=modified Rankin Scale.

Supplementary Table S4. Peripheral blood biomarkers in the overall study population and by collateral status

	All Patients<br>n = 105	Good<br>n = 44	Moderate<br>n = 36	Poor<br>n = 22	p-value
**Admission (T0)**					
Onset-to-blood-sample time (min), median (IQR)	120.0<br>(80-198.5)	105.0<br>(65-190)	135.0<br>(90-240)	90.0<br>(75.0-180.0)	0.609
Total leukocyte (10³/µL)					
- Mean (SD)	8.77 (2.76)	8.54 (2.47)	8.30 (2.43)	9.54 (3.10)	0.205
- Median (IQR)	8.10<br>(6.89-10.33)	7.87<br>(6.65-10.08)	7.92<br>(6.59-9.33)	9.07<br>(7.02-11.36)	
Neutrophils (10³/µL),					
absolute count	6.23 (2.61)	6.04 (2.27)	5.98 (2.46)	6.46 (2.58)	0.740
- Mean (SD)	5.54	5.35	5.98	5.74	
- Median (IQR)	(4.61-7.34)	(4.38-7.12)	(4.25-7.15)	(4.87-8.31)	
Neutrophils (%), percentage					
- Mean (SD)	69.77 (11.51)	69.98 (10.64)	70.19 (12.41)	67.29 (11.23)	0.603
- Median (IQR)	70.60<br>(61.33-79.0)	70.40<br>(62.10-77.90)	70.90<br>(61.50-79.20)	64.55<br>(57.58-78.38)	
Lymphocytes (10³/µL), absolute count					
- Mean (SD)	1.78 (1.02)	1.75 (0.90)	1.63 (1.02)	2.18 (1.18)	0.128
- Median (IQR)	1.56<br>(1.19-2.11)	1.56<br>(1.09-2.13)	1.47<br>(1.08-1.93)	2.0<br>(1.33-2.93)	
Lymphocytes (%),					
percentage	20.77 (9.93)	21.17 (9.49)	19.59 (10.41)	23.10 (9.84)	0.430
- Mean (SD)	20.20	21.10	17.80	25.45	
- Median (IQR)	(12.60-27.08)	(13.75-27.08)	(11.70-26)	(15.78-30.13)	
PCR (mg/dL)					
- Mean (SD)	1.87 (2.79)	1.91 (0.17)	1.38 (1.80)	2.49 (3.34)	0.526
- Median (IQR)	0.42<br>(0.12-2.42)	0.17<br>(0.10-2.03)	0.48<br>(0.17-2.08)	0.42<br>(0.15-6.55)	
NO (µM)					
- Mean (SD)	49.71 (19.34)	48.36 (20.33)	50.80 (18.94)	50.64 (19.22)	0.860
- Median (IQR)	48.34<br>(37.23-61.85)	47.82<br>(29.81-59.0)	52.17<br>(38.07-65.23)	49.26<br>(39.34-60.46)	
ET-1 (pg/dL)					
- Mean (SD)	11.93 (5.68)	10.80 (4.51)	12.81 (6.61)	13.55 (6.26)	0.182
- Median (IQR)	10.74<br>(8.36-13.89)	10.43<br>(7.96-13.38)	10.67<br>(8.63-16.0)	12.42<br>(8.86-17.56)	
24 h (T1)					
Onset-to-blood-sample time, hours, median (IQR)	25.75<br>(25-27.75)	25.63<br>(25-27.36)	26.25<br>(25.37-29.50)	25.50<br>(24.50-27.0)	0.925
Total leukocyte (10³/µL)					
- Mean (SD)	10.19 (3.60)	9.61 (3.34)	10.13 (3.83)	11.28 (3.32)	0.368
- Median (IQR)	9.15<br>(7.61-12.33)	8.93<br>(7.35-12.28)	8.79<br>(7.82-12.64)	10.72<br>(8.80-14.75)	
Neutrophils (10³/µL),					
absolute count	8.29 (3.63)	7.75 (3.35)	8.3 (3.94)	9.12 (3.31)	0.509
- Mean (SD)	7.64	7.21	7.41	8.57	
- Median (IQR)	(5.69-10.35)	(5.29-10.15)	(5.62-10.03)	(6.82-12.78)	
Neutrophils (%), percentage					

- Mean (SD)	78.8 (10.0)	77.37 (10.89)	80.09 (9.79)	79.30 (7.61)	0.600
- Median (IQR)	81.20	79.60	82.30	82.10	
	(72.18-87.03)	(70.53-87.63)	(72.70-87.10)	(74.58-84.70)	
Lymphocytes (10^3/μL),					
absolute					
count	1.22 (0.56)	1.26 (0.61)	1.11 (0.55)	1.32 (0.48)	0.543
- Mean (SD)	1.12	1.10	0.92	1.32	
- Median (IQR)	(0.79-1.55)	(0.75-1.69)	(0.80-1.39)	(1.04-1.74)	
Lymphocytes (%),					
percentage	13.69 (7.99)	14.64 (8.55)	12.94 (8.03)	12.91 (6.49)	0.686
- Mean (SD)	10.95	13.65	10.50	11.25	
- Median (IQR)	7.43-19.93)	(7.28-20.70)	(7.85-19.20)	(7.83-18.90)	
PCR (md/dL)					
- Mean (SD)	2.05 (2.17)	1.91 (2.37)	2.14 (2.11)	1.72 (1.45)	0.884
- Median (IQR)	1.31	1.09	1.22	1.68	
	(0.53-2.77)	(0.55-1.85)	(0.30-3.68)	(0.69-1.99)	
NO (μM)					
- Mean (SD)	23.46 (12.16)	21.54 (13.37)	24.79 (9.68)	27.44 (12.79)	0.239
- Median (IQR)	24.26	20.20	25.52	31.62	
	(12.93-32.12)	(8.76-31.54)	(18.11-29.65)	(16.40-36.71)	
ET-1 (pg/dL)					
- Mean (SD)	16.63 (8.18)	16.42 (10.03)	16.96 (6.39)	17.20 (7.35)	0.941
- Median (IQR)	15.38	15.21	16.02	14.55	
	(11.42-20.84)	(9.97-20.68)	(11.90-20.67)	(11.51-23.66)	
48 h (T2)					
Onset-to-blood-sample	50.5	49.9	50.0	49.40	0.540
time, hours, median (IQR)	(49-52)	(49-51-63)	(49.10-53.50)	(48.63-51.0)	
Total leukocyte					
- Mean (SD)	10.37 (4.59)	9.42 (3.26)	10.06 (5.24)	11.98 (4.69)	0.202
- Median (IQR)	9.39	8.71	9.16	9.79	
	(7.59-11.92)	(7.32-11.28)	(7.56-11.67)	(8.81-14.24)	
Neutrophils (10^3/μL),					
absolute count	8.27 (4.50	7.47 (3.21)	7.88 (5.32)	9.77 (4.36)	0.252
- Mean (SD)	7.13	6.58	6.89	8.18	
- Median (IQR)	(5.70-9.82)	(5.36-8.58)	(5.18-9.39)	(6.85-12.23)	
Neutrophils (10^3/μL),					
percentage	77.18 (11.94)	75.10 (15.40)	77.26 (10.38)	80.20 (5.88)	0.422
- Mean (SD)	78.40	76.90	78.40	80.60	
- Median (IQR)	(73.63-85.05)	(72.70-82-70)	(70.85-86.30)	(75.10-85.30)	
Lymphocytes (10^3/μL),					
absolute					
count	1.43 (1.31)	1.19 (0.51)	1.52 (1.60)	1.68 (1.77)	0.478
- Mean (SD)	1.12	1.04	1.25	1.13	
- Median (IQR)	(0.82-1.56)	(0.82-1.44)	(0.79-1.65)	(0.83-1.83)	
Lymphocytes (%),					
percentage	13.49 (7.43)	13.83 (6.41)	14.48 (9.30)	11.54 (5.41)	0.471
- Mean (SD)	12.20	13.10	11.50	10.70	
- Median (IQR)	(8.15-16.55)	(9.20-17.90)	(7.16-19.68)	(7.90-15.30)	
PCR (mg/dL)					
- Mean (SD)	4.59 (5.43)	2.77 (3.11)	6.69 (8.09)	4.90 (3.55)	0.082
- Median (IQR)	2.75	1.67	3.79	4.25	
	(1.01-6.19)	(0.92-4.11)	(1.17-9.80)	(2.35-7.19)	

NO (μM)					
- Mean (SD)	16.43 (8.65)	14.81 (8.48)	18.71 (8.87)	17.71 (7.54)	0.167
- Median (IQR)	15.25 (10.0-22.90)	14.06 (6.54-22.12)	15.92 (11.93-24.08)	17.40 (13.22-23.28)	
ET-1 (pg/dL)					
- Mean (SD)	16.06 (6.51)	16.17 (6.56)	16.85 (5.92)	14.53 (8.06)	0.532
- Median (IQR)	15.31 (11.49-20.38)	17.34 (11.47-21.74)	16.21 (12.13-20.77)	13.24 (8.22-18.85)	

T0 = time point 0; min = minutes; IQR = interquartile range; $10^3/\mu L$ = thousand per microliters; SD = standard deviation; PCR = C-reactive protein; mg/dL = milligrams per deciliter; NO = nitric oxide; μM = micromoles; ET-1 = endothelin-1; pg/dL = picograms per deciliter; T1 = time point 1; T2 = time point 2.
Section headings are formatted in bold to distinguish different categories of data within the table.

Supplementary Table S5. Intracranial blood biomarkers (pre- and post-occlusion) in the overall study population and by collateral status

	**All Patients** n = 105	**Good** n = 44	**Moderate** n = 36	**Poor** n = 22	**p-value**
**Admission (T0)**					
NO (μM)					
Pre-occlusion	n = 19	n = 7	n = 8	n = 4	
- Mean (SD)	47.60 (8.80)	48.62 (9.42)	46.17 (10.09)	48.69 (6.35)	0.848
- Median (IQR)	47.69 (40.94-55.22)	44.50 (40.94-58.43)	47.02 (36.13-55.22)	50.55 (42.0-53.53)	
Post-occlusion	n = 5	n = 2	n = 2	n = 1	
- Mean (SD)	39.48 (12.81)	43.68 (20.03)	42.25 (3.28)	25.56	-
- Median (IQR)	39.93 (27.54-51.21)	-	-	-	
ET-1 (pg/dL)					
Pre-occlusion	n = 39	n = 21	n = 12	n = 6	
- Mean (SD)	9.60 (5.46)	9.10 (4.16)	11.39 (7.70)	7.77 (3.76)	0.354
- Median (IQR)	8.86 (5.23-11.50)	8.86 (6.78-10.0)	9.78 (4.77-19.17)	8.75 (3.30-11.17)	
Post-occlusion	n = 5	n = 2	n = 2	n = 1	
- Mean (SD)	3.56 (0.30)	3.42 (0.30)	3.64 (0.45)	3.67	-
- Median (IQR)	3.63 (3.27-3.82)	-	-	-	

T0 = time point 0; NO = nitric oxide; μM = micromoles; SD = standard deviation; IQR = interquartile range; ET-1 = endothelin-1; pg/dL = picograms per deciliter.
Section headings are formatted in bold.
